# Characterization and Selective Antiglioma Activity of a Standardized Hydroalcoholic Extract from Yellow *Psidium cattleianum* Fruits

**DOI:** 10.1007/s11064-026-04907-3

**Published:** 2026-10-08

**Authors:** Francieli da Silva dos Santos, Juliane Torchelsen Saraiva, Giulia Bueno de Oliveira da Silva, Natália Pontes Bona, William Sanabria Simões, Jessica Fernanda Hoffmann, Fabio Clasen Chaves, Marcia Vizzotto, Gabriela Nogueira Debom, Elizandra Braganhol, Mayara Sandrielly Soares de Aguiar, Roselia Maria Spanevello, Nathalia Stark Pedra, Francieli Moro Stefanello

**Affiliations:** 1 Programa de Pós-Graduação em Bioquímica e Bioprospecção, Universidade Federal de Pelotas, Capão do Leão, 96160-000 RS Brazil; 2 Instituto Tecnológico em Alimentos para a Saúde, Universidade do Vale do Rio dos Sinos, São Leopoldo, 93022-750 Brazil; 3 Programa de Pós-Graduação em Ciência e Tecnologia de Alimentos, Universidade Federal de Pelotas, Capão do Leão, 96160-000 RS Brazil; 4 Empresa Brasileira de Pesquisa Agropecuária Clima Temperado, Pelotas, 96010-971 RS Brazil; 5 Programa de Pós-Graduação em Biociências, Universidade Federal de Ciências da Saúde de Porto Alegre, Porto Alegre, 90050-170 RS Brazil

**Keywords:** Natural products, Cancer, Glioblastoma, Ellagic acid, Apoptosis, Cytotoxicity

## Abstract

Glioblastoma (GBM) is an aggressive and prevalent malignant brain tumor and remains a major public health challenge. Prognosis is poor, with median survival of 14–18 months and fewer than 7% of patients surviving beyond five years, reinforcing the need for new therapeutic strategies. Natural products are a traditional source of bioactive substances, and fruits are rich in phenolic constituents with potential anticancer activity. *Psidium cattleianum*, a Myrtaceae species native to South America, produces red and yellow fruits rich in vitamin C and phenolics, although biological data on the yellow variety are scarce. Preliminary evidence suggests anti-inflammatory, antioxidant, and antineoplastic activities, but studies in glioma models are limited. This study characterized the phenolic profile and evaluated the activity of a hydroalcoholic extract from yellow *Psidium cattleianum* (YPC) fruits in GBM and non-tumor cells. LC-MS analysis identified ellagic acid, catechin, and gallic acid as major constituents. Biological activity was assessed in glioma cell lines (C6 and U87MG), primary astrocytes, and fibroblast lines (L929 and MRC5). Cells were treated with extract concentrations ranging from 125 to 2000 µg/mL for 2 to 72 h. The extract significantly reduced proliferation, viability, colony formation and reactive oxygen species levels in glioma cells, while increasing glutathione peroxidase activity. In C6 cells, it also inhibited migration. Astrocytes and fibroblasts remained unaffected by cytotoxicity under the evaluated conditions. These findings demonstrate a selective antineoplastic potential of the YPC extract in glioma cells and support its relevance for further investigation in GBM treatment.

**Graphical Abstract:**

**Figure:**
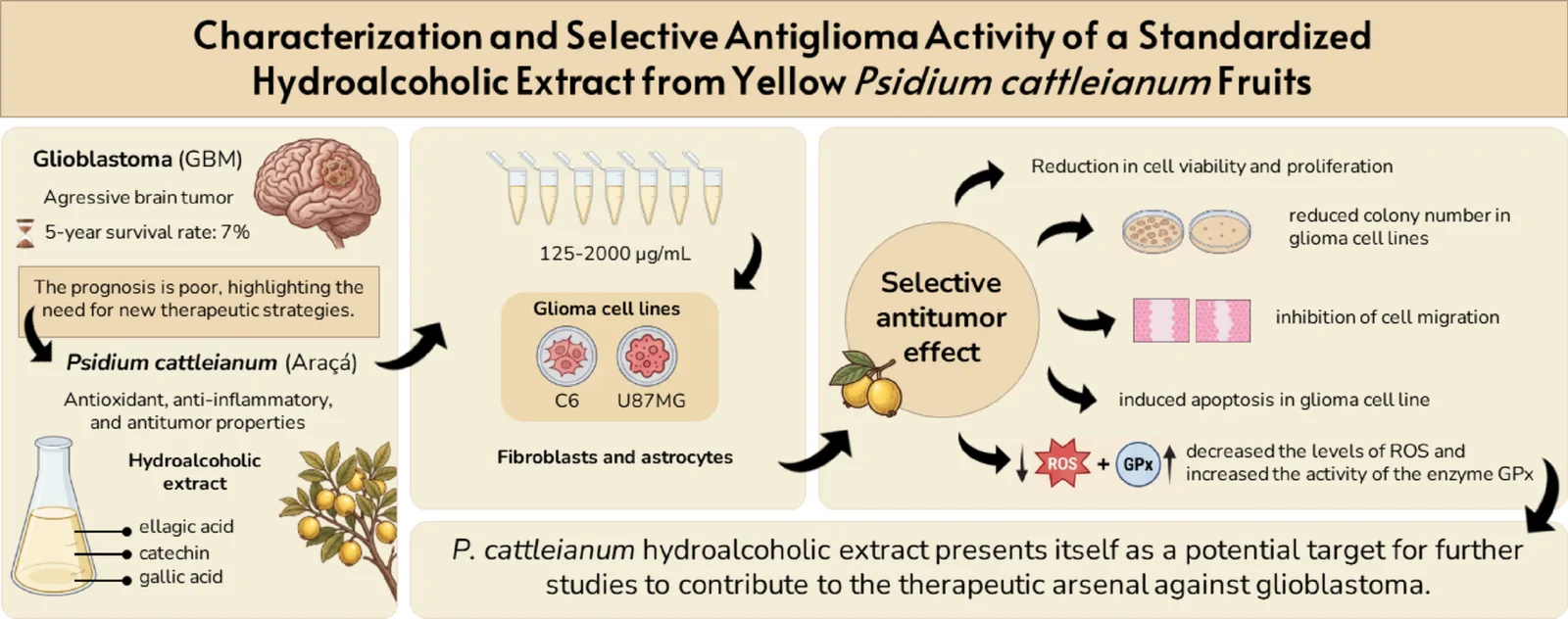

## Introduction

A defining public health challenge of the current century is the global prevalence of cancer, accounting for three out of every ten premature deaths among individuals aged 30–69 years [1]. When occurring intracranially, it carries steep lethality risks, primarily driven by its location and the invasive growth it frequently exhibits [2]. Of all cerebral tumors, 26.3% display malignant behavior, and 22.2% comprise gliomas, therefore representing the most common type of malignant brain tumor [3].

Glioblastoma (GBM) is the most prevalent and aggressive type of glioma, which can be classified as an adult grade 4 glioma and accounts for 61.7% of gliomas [3]. Despite treatment, the estimated median survival for patients is only 14–18 months, and only 7% survive five years or longer [3, 4]. The standard treatment approach consists of maximal safe surgical resection, radiotherapy, and chemotherapy with temozolomide (TMZ) [4]. However, this approach presents several challenges, including emerging resistance to TMZ, the restrictive nature of the blood-brain barrier, and tumor characteristics such as cellular heterogeneity, diffuse brain infiltration, and rapid proliferative capacity, all of which reflect the malignant nature of GBM and compromise treatment efficacy and patient outcomes [5, 6].

Another characteristic of malignancy is the ability to proliferate even under conditions of redox imbalance [7, 8]. This imbalance can be characterized as a disruption between antioxidants and oxidants, such as reactive oxygen species (ROS) [7]. Owing to its high energy consumption, the brain is highly vulnerable to oxidative damage, potentially leading to oncogenesis [7]. Furthermore, ROS-mediated cellular signaling plays a major role in key aspects of tumor progression, including proliferation, growth, angiogenesis, and survival [7–9]. Given the malignancy features, the limitations of current therapy, and the poor patient outcomes, exploring novel therapeutic alternatives capable of improving both prognosis and quality of life in patients with GBM is crucial.

Natural products are noteworthy, having historically paved the way for novel drugs [10]. Between 1981 and 2019, close to 32% among approved drugs were derived from natural sources [11]. Nearly 65% of the global population uses natural bioactive compounds as a primary strategy for managing various disorders [10]. Accordingly, plant and fruit extracts remain promising candidates for evaluation as potential therapeutics against GBM.

The fruits of *Psidium cattleianum* are a worthwhile subject for study. A Myrtaceae species native to South America, it is easily cultivated in tropical and subtropical regions and is now distributed across multiple countries worldwide [12, 13]. The plant produces small fruits of varying colors, all with succulent pulp rich in seeds and high levels of vitamin C and phenolic compounds.

The yellow fruits, known as yellow araçá, yellow guava, yellow strawberry guava, or yellow cherry guava, exhibit anti-inflammatory, antioxidant, and antineoplastic activities [13–16]. However, their biological effects remain poorly studied, and only one study has evaluated activity in a glioma model, focusing exclusively on antiproliferative effects [17]. Therefore, this study investigates the phenolic profile and antitumor effects of a hydroalcoholic extract from yellow *Psidium cattleianum* (YPC) fruits in GBM and non-tumor cells.

## Materials and methods

### Hydroalcoholic Extract from Yellow *Psidium cattleianum* Fruits

#### Fruits and Preparation of the Extract

YPC fruits were obtained from the Brazilian Agricultural Research Corporation, Temperate Agriculture, Pelotas, RS, Brazil (31°40’47"S, 52°26’24"W) during the 2018 harvest season. Fruits were selected at full commercial maturity based on visual criteria (uniform skin coloration and absence of physical damage). Immediately following harvest, fruits were frozen and stored at -20 °C. Whole fruit samples, including flesh, peel, and seeds, were chopped and homogenized together to form a composite sample. An aliquot of 80 g of this mixture was extracted in an ultrasonic bath for 30 min at 25 °C under light-protected conditions using a 70:30 (v/v) mixture of absolute ethanol and distilled water (240 mL). The extract was subsequently filtered, ethanol was removed under reduced pressure (36–37 °C), and the resulting product was lyophilized and stored at -80 °C [18].

#### LC-MS Analysis and Quantification of Phenolic Content

The phenolic content was investigated according to Ramos et al. [18]. The lyophilized hydroalcoholic extract was resuspended in 75% (v/v) methanol (final concentration 57.5 mg/mL) and filtered through a nylon membrane (0.22 μm) prior to analysis. LC-MS analysis was performed on a Prominence UFLC system (Shimadzu, Japan) coupled to an Impact HD mass spectrometer (Bruker Daltonics, Germany). Metabolites were separated using a Bidentate C18 column (100 × 2.1 mm, 2.2 µM, 100Å, MicroSolv Technology Corporation, USA). Mobile phases consisted of 0.1% aqueous formic acid (pH 2.8; solvent A) and acetonitrile (solvent B). The gradient program was: 5% B at 0 min, increased linearly to 90% B at 15 min, held for 3 min at 90% B, returned to 5% B over 2 min, and maintained for 6 min at a flow rate of 0.2 mL/min. The injection volume was 10 µL.

MS parameters were set in negative ionization mode with spectra acquired over *m/z* 50-1200. Parameters were: capillary voltage, 3.5 kV; drying gas temperature, 180 °C; drying gas flow, 8.0 L/min; nebulizing gas pressure, 2 bar; collision RF, 300 Vpp; transfer time, 120 µs; prepulse storage, 8 µs. Collision energy was automatically adjusted for MS/MS experiments according to *m/z*: 100, 15 eV; 500, 35 eV; 1000, 50 eV, using nitrogen as collision gas.

For quantification of phenolic acids and flavonoids, external calibration curves (39–5,000 ng/mL) with analytical standards (Sigma-Aldrich) were prepared. The monitored ions in the negative mode were: [M-H]⁻ *m/z* 169.0132 (gallic acid, Rt = 2.27 min, y = 135.54x − 3,486, R² = 0.9999), *m/z* 179.0350 (caffeic acid, Rt = 8.47 min, y = 211.10x − 4,190, R² = 0.9999), *m/z* 163.0401 (*p*-coumaric acid, Rt = 9.32 min, y = 185.96x + 7,616, R² = 0.9993), *m/z* 353.0867 (chlorogenic acid, Rt = 13.63 min, y = 120.45x − 6,875, R² = 0.9996), *m/z* 300.9978 (ellagic acid, Rt = 15.61 min, y = 138.58x − 4,792, R² = 0.9990), *m/z* 285.0393 (kaempferol, Rt = 17.68 min, y = 907.02x − 2,889, R² = 0.9992), *m/z* 301.0354 (quercetin, Rt = 16.85 min, y = 370.37x + 1,689, R² = 0.9947), and *m/z* 289.0718 (catechin, Rt = 7.79 min, concentration range: 39 − 2,500 ng/mL, y = 54.33x + 325, R² = 0.9989). Results are expressed as µg/g dry weight of lyophilized extract, mean ± standard deviation of four replicates.

### Cell Culture

Cell lines used were C6 (rat glioma), U87MG (human glioma), L929 (mouse fibroblast), and MRC5 (human fibroblast), all purchased from the American Type Culture Collection (ATCC, USA). Cells were cultured in Dulbecco’s Modified Eagle Medium (DMEM, pH 7.4; Gibco, USA) supplemented with amphotericin B (0.1% v/v; Gibco, USA), penicillin/streptomycin (0.5% v/v; Gibco, USA), and fetal bovine serum (FBS, 10% v/v; Gibco, USA). MRC5 medium was additionally supplemented with glucose at 0.35% (m/v). Incubation was performed under standard conditions (37 °C, 5% CO₂, humidified atmosphere). Cells were distributed into 96-well plates at 5 × 10³ cells/well for the 3-(4,5-dimethylthiazol-2-yl)-2,5-diphenyltetrazolium bromide (MTT; Sigma-Aldrich, USA) and sulforhodamine B (SRB; Sigma-Aldrich, USA) assays. For migration and clonogenic assays, cells were plated in 24-well plates at 2 × 10⁴ cells/well, and for cell death analysis, C6 cells were established in 12-well plates at 1 × 10⁵ cells/well.

### Primary Astrocyte Culture

The primary astrocyte cultures were established as previously described by our research group [19]. Culture plates were initially coated with poly-L-lysine to facilitate cell attachment. Cerebral cortices were obtained from Wistar rat pups aged 1–2 days. The animals were euthanized by decapitation using sterile, autoclaved surgical scissors inside a laminar flow cabinet, separate from the cabinet subsequently used for cell culture procedures. Following euthanasia, the animal heads were transferred to the previously sanitized and sterilized laminar flow hood. The cabinet was disinfected with 70% ethanol and ultraviolet light, and all materials required for tissue collection and processing were previously sterilized by autoclaving and handled exclusively within the laminar flow environment.

The brains were rapidly removed, and the meninges were carefully discarded. The cerebral cortex was then isolated and mechanically dissociated in calcium- and magnesium-free balanced salt solution (CMF, pH 7.4). Tissue disruption was achieved by repeated and gentle trituration using a pipette until a homogeneous cell suspension was obtained. The suspension was centrifuged at 1000 × g for 10 min, after which the resulting cell pellet was resuspended in DMEM containing 10% FBS (pH 7.6). An antibiotic-antifungal supplement was incorporated into the culture medium to reduce the risk of bacterial and fungal contamination.

The resulting cell suspension was plated in 96-well culture plates. Four hours after plating, the cultures were gently washed to remove non-adherent material, and fresh supplemented medium was added. Cells were maintained at 37 °C in a humidified atmosphere containing 5% CO₂. The culture medium was replaced every four days, and the cells were maintained for up to 20 days. Confluence was generally reached between days 15 and 20 of culture, providing a suitable period for cellular differentiation and establishment of a stable primary cortical cell culture for subsequent experiments.

The study was approved by the Animal Experimentation Ethics Committee of the Federal University of Pelotas (protocol CEEA 31292 − 2018) and complied with the Brazilian Guidelines for the Care and Use of Research Animals in Sciences and the regulations of the National Council for Animal Control and Experimentation.

### Cell Treatment

The YPC extract was diluted in sterile water to 10 mg/mL and further diluted with DMEM to obtain concentrations of 125, 250, 500, 750, 1000, 1500, and 2000 µg/mL, based on a previous study [20]. Tumor cell lines (C6 and U87MG) were treated with the extract (125–2000 µg/mL) for 24, 48, and 72 h. MRC5, L929, and primary astrocytes were treated for 48 h with the highest concentrations (500–2000 µg/mL). In the clonogenic assay, C6 and U87MG cells were exposed to 1500 and 2000 µg/mL, respectively, and in the migration assay, C6 cells were treated with 750 µg/mL (0–24 h). For cell death analysis, C6 cells were incubated with 750–2000 µg/mL. Untreated cells served as controls.

### Cell Viability Assay

Cell viability was assessed using the MTT assay as described by Mosmann [21]. This colorimetric method relies on the ability of viable cell mitochondria to reduce MTT to blue formazan crystals. Following treatment with the extract, the wells were rinsed with CMF and incubated with MTT (0.5 mg/mL) under standard culture conditions for 90 min. The resulting formazan precipitate was dissolved in 50 µL of dimethyl sulphoxide (DMSO; Sigma-Aldrich, USA), and optical density (OD) was measured at 492 nm using a microplate reader (SpectraMax 190; Molecular Devices, USA). Cell viability was expressed as a percentage relative to the control: cell viability rate (%) = (OD_492_ of extract-treated cells/OD_492_ of control) × 100%. This assay was performed in technical triplicate within an independent experiment.

### Cell Proliferation Assay

Proliferation was evaluated utilizing the SRB assay as previously described [22]. This method relies on SRB binding to cellular proteins in an acidic medium. Post-treatment, the cells were fixed with 50 µL of 50% trichloroacetic acid (TCA; Sigma-Aldrich, USA) for 45 min at 4 °C. Wells were then washed with distilled water to remove residual TCA. Cells were stained with 50 µL of SRB solution (0.4% in 1% acetic acid) for 30 min. Excess dye was removed by washing with 1% acetic acid, and 50 µL of 10 mM Tris solution was added to elute the bound dye. Optical density (OD) was measured at 530 nm using a microplate reader (SpectraMax 190; Molecular Devices, USA). Cell proliferation was expressed as a percentage relative to the control: cell proliferation rate (%) = (OD_530_ of extract-treated cells/OD_530_ of control) × 100%. This assay was performed in technical triplicate within an independent experiment.

### Clonogenic Assay

Cell survival was evaluated using clonogenic analysis, which determines the capacity of individual cells to proliferate into colonies [23]. Tumor cell lines were exposed to 1500 µg/mL (C6) and 2000 µg/mL (U87MG) of the YPC extract for 48 h. Higher concentrations were selected based on preliminary cytotoxicity screening to capture threshold levels capable of inducing persistent growth inhibition after treatment withdrawal. Treated cells were seeded in 6-well plates at 300 cells per well and maintained under standard conditions for 10 days. Cells were subjected to fixation using ice-cold absolute methanol (Sigma-Aldrich, USA) and a 1% crystal violet solution (Sigma-Aldrich, USA) was applied for staining. Microscopic evaluation of colony number (clonogenic efficiency) and size (proliferation dynamics and growth kinetics of surviving cells over time) was assessed using an inverted microscope (40×) coupled to a digital camera (Olympus EP50; Olympus, Japan) and ImageJ 1.51j8 software (National Institutes of Health, USA). This assay was performed in technical triplicate within an independent experiment.

### Cell Migration Assay

Cell migration was assessed following a scratch in a confluent monolayer, in accordance with the protocol by Saraiva et al. [20]. To induce the wound, a sterile 200 µL pipette tip was used. Cells were then washed with CMF and treated with YPC extract (750 µg/mL). Microscopic observation of the wound area occurred at 0, 2, 4, 6, and 24 h using an inverted microscope (10×) equipped with an Olympus EP50 digital imaging system (Olympus EP50; Olympus, Japan). The cell-free area at each time point was quantified using ImageJ software (National Institutes of Health, USA), and the results were calculated and expressed as the percentage of cell migration relative to the control group. This assay was performed in technical triplicate within an independent experiment.

### Cell Death Analysis

Apoptosis and necrosis were assessed in C6 cells using Annexin V-FITC (AnV; BD Biosciences, USA) and propidium iodide (PI; Sigma-Aldrich, USA) according to Bona et al. [24]. After 72 h of treatment, media were collected, and cells were trypsinized and washed with cold phosphate-buffered saline (PBS, pH 7.4). Suspended and adherent cells were combined to include both dead and living populations. A total of 5 × 10⁵ cells were resuspended in 100 µL of Annexin Binding Buffer (ABB; BD Biosciences, USA) with 2 µL FITC-conjugated Annexin V and 3 µL PI, followed by 15 min incubation. Subsequently, 400 µL of ABB was added before flow cytometry analysis. AnV and PI expression were measured using a BD Symphony A5 flow cytometer (BD Biosciences, USA), and data were analyzed with FlowJo software (BD Biosciences, USA). Results are expressed as the percentage of AnV- or PI-positive cells. Cells incubated for 5 min at 60 °C in a water bath served as a positive control for both AnV and PI. This assay was performed in technical triplicate within an independent experiment.

### Redox Balance Parameters

#### Sample Preparation and Protein Determination

Cell lysates were prepared as previously reported by Simões et al. [25]. After 48 h of treatment with YPC extract (500–2000 µg/mL) in C6 and U87MG cell lines, the culture medium was removed and the cells were washed twice with sterile water. Subsequently, cells were detached with a cell scraper and mechanically lysed. After centrifugation at 1000 × g for 10 min, the supernatant was collected and stored at -80 °C. This lysate was used to investigate antioxidant enzyme activities. To assess redox balance parameters, proteins were quantified according to Lowry et al. [26].

#### ROS Levels

To determine intracellular ROS levels, the methodology proposed by Dos Santos et al. [27] was used, which is based on the oxidation of 2’,7’-dichlorodihydrofluorescein diacetate (DCFH-DA) to the fluorescent dichlorofluorescein (DCF). After 48 h of treatment with YPC extract (500–2000 µg/mL) in C6 and U87MG cell lines, the medium was removed, and 190 µL of PBS and 10 µL of DCFH-DA were added under dark conditions. The plates were incubated for 30 min at 37 °C, and fluorescence was measured in a microplate reader (kinetic mode, every 30 s for 5 min) at 480 and 520 nm (SpectraMax 190). ROS levels were expressed as percentages of the control. This assay was performed in technical triplicate within an independent experiment.

#### Superoxide Dismutase (SOD) Activity

SOD activity was assessed based on its ability to inhibit the superoxide-dependent autoxidation of epinephrine, as detailed by Misra & Fridovich [28]. The assay mixture contained 10 µL of cell lysate, 10 µM catalase, 50 mM glycine buffer (pH 10.2), and 60 mM adrenaline. Absorbance was monitored at 480 nm using a SpectraMax 190 microplate reader. SOD activity was expressed as a percentage of the control. This assay was performed in technical triplicate within an independent experiment.

#### Catalase (CAT) Activity

CAT activity was determined by measuring hydrogen peroxide (H₂O₂) decomposition, following the protocol described by Aebi [29]. Briefly, a 30 µL of cell lysate was mixed with Triton X-100 (1:10 w/v). The reaction was initiated by adding 30 mM H₂O₂ in a 50 mM potassium phosphate buffer. Absorbance changes were monitored at 240 nm for 180 s at 37 °C using a SpectraMax 190 microplate reader. CAT activity was expressed as a percentage of the control. This assay was performed in technical triplicate within an independent experiment.

#### Glutathione Peroxidase (GPx) Activity

GPx activity was determined using the RANSEL kit produced by RANDOX (Randox Laboratories Ltd., UK). Absorbance changes were monitored at 340 nm for 120 s at 37 °C using a SpectraMax 190 microplate reader. GPx activity was expressed as a percentage of the control. This assay was performed in technical triplicate within an independent experiment.

### Statistical Analysis

All assays were performed in triplicate within three independent experiments. Statistical analyses were conducted using Student’s t-test or one-way/two-way ANOVA with Tukey’s or Bonferroni’s *post-hoc* tests, as appropriate. Analyses were performed using GraphPad Prism version 9.5.0 (GraphPad Software, USA). A *p* value < 0.05 was considered statistically significant.

## Results

### Phenolic Content of Yellow *Psidium cattleianum* Extract

Table 1 presents the phenolic content of the hydroalcoholic extract from YPC fruits. A total of eight individual phenolic compounds were identified and quantified. These include the phenolic acids caffeic, chlorogenic, gallic, and *p*-coumaric; the flavonoids (+)-catechin, kaempferol, and quercetin; and the hydrolyzable tannin derivative ellagic acid. Ellagic acid was the predominant compound (13.34 ± 0.39 µg g⁻¹), followed by catechin (6.42 ± 0.06 µg g⁻¹) and gallic acid (4.47 ± 0.10 µg g⁻¹).

### Yellow *Psidium cattleianum* Extract Selective Cytotoxic Effect Against Glioma Cells

**Fig. 1:**
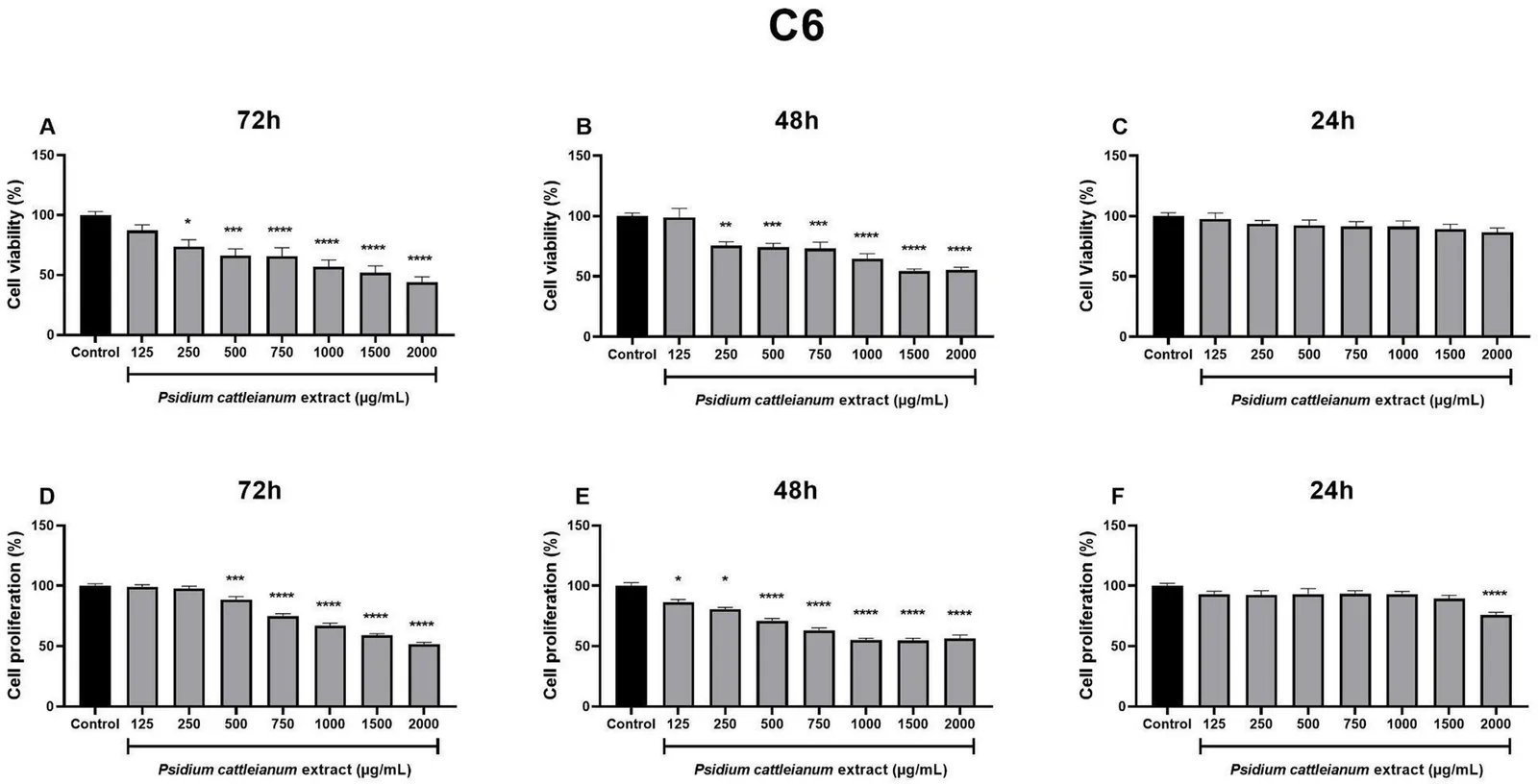
Effect of hydroalcoholic extract from yellow *Psidium cattleianum* fruits on cell viability **A**–**C** and cell proliferation **D**–**F** in C6 rat glioma cells at 72 h **A** and **D**, 48 h **B** and **E**, and 24 h **C** and **F**. Data are expressed as mean ± standard error and analyzed by one-way ANOVA with Tukey’s *post-hoc* test. *p < 0.05, **p < 0.01, ***p < 0.001, ****p < 0.0001 significantly different from the control. Three independent experiments were performed in triplicate

**Fig. 2:**
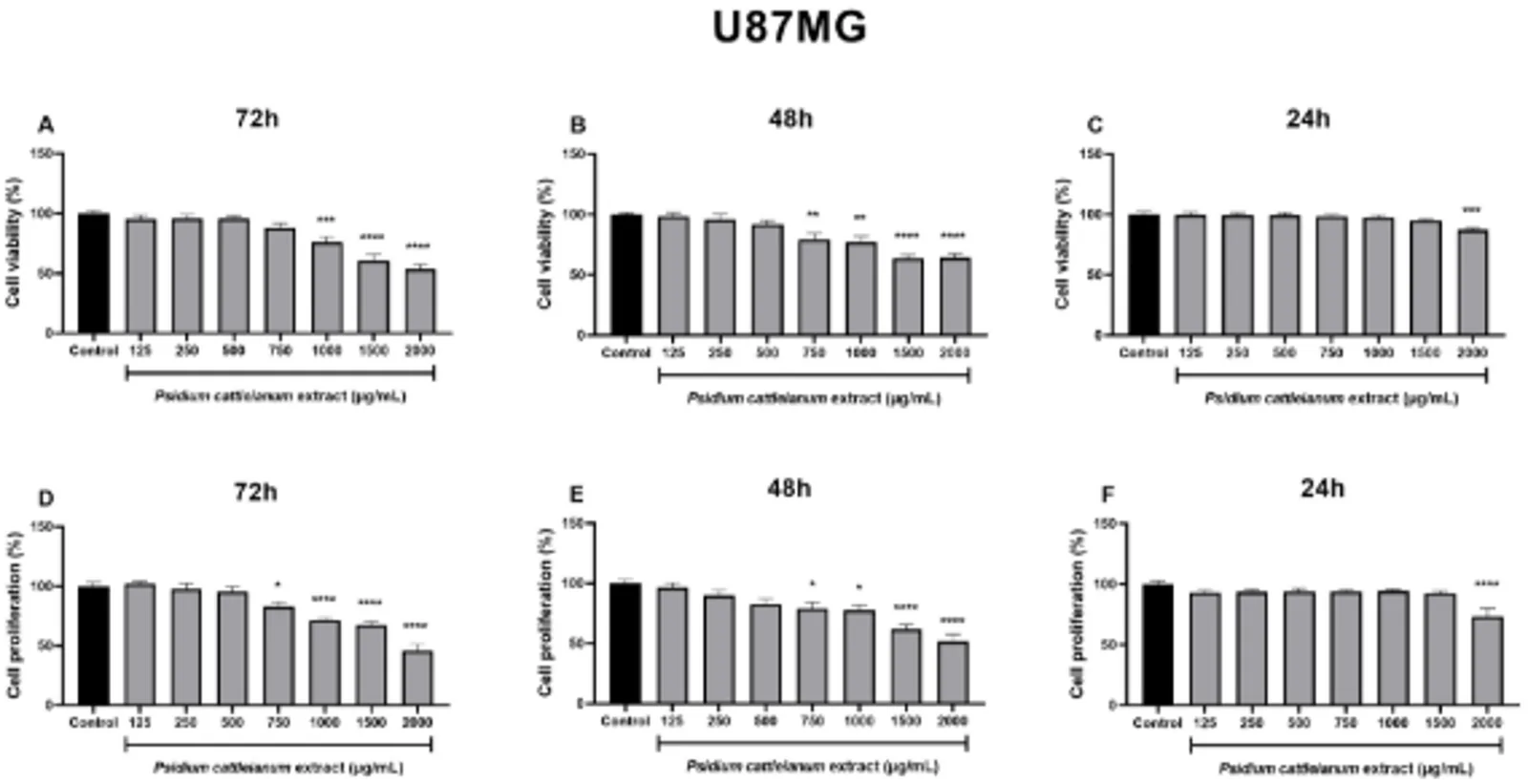
Effect of hydroalcoholic extract from yellow *Psidium cattleianum* fruits on cell viability **A**–**C** and cell proliferation **D**–**F** in U87MG human glioma cells at 72 h **A** and **D**, 48 h **B** and **E**, and 24 h **C** and **F**. Data are expressed as mean ± standard error and analyzed by one-way ANOVA with Tukey’s *post-hoc* test. *p < 0.05, **p < 0.01, ***p < 0.001, ****p < 0.0001 significantly different from the control. Three independent experiments were performed in triplicate

As shown in Figs. 1 and 2, YPC extract exhibited a concentration-dependent cytotoxic effect against both glioma cells (C6 and U87MG). After 72 h, treatment with the extract at concentrations of 500, 750, 1000, 1500, and 2000 µg/mL reduced the viability of C6 cells by 33.9%, 34.3%, 43.3%, 48.2%, and 55.9%, respectively, compared to the control (Fig. 1A). Similarly, cell proliferation was reduced at concentrations ranging from 500 to 2000 µg/mL, by 11.4%, 24.9%, 33.0%, 41.0%, and 48.3%, respectively, compared to the control (Fig. 1D). After 48 h, a reduction in viability ranging from 24.4 to 45.4% (at concentrations of 250–2000 µg/mL) was observed (Fig. 1B), and a decrease in cell proliferation ranging from 13.4 to 45.2% (at concentrations of 125–2000 µg/mL) was noted (Fig. 1E). At 24 h, only cell proliferation was significantly reduced (Fig. 1F).

Reductions in U87MG cell viability of 23.9%, 39.3%, and 46.3% (1000–2000 µg/mL) and in cell proliferation of 16.9%, 28.3%, 32.5%, and 54.4% (750–2000 µg/mL) were observed after 72 h of treatment (Fig. 2A and D). After 48 h, a reduction in cell viability and proliferation was observed at concentrations starting from 750 µg/mL of the extract, with reductions ranging from 21.0 to 35.8% for viability and 21.1–48.1% for proliferation, respectively (Fig. 2B and E). At 24 h, a cytotoxic effect was observed against the U87MG cell line at the highest tested extract concentration (2000 µg/mL) (Fig. 2C and F).

**Fig. 3:**
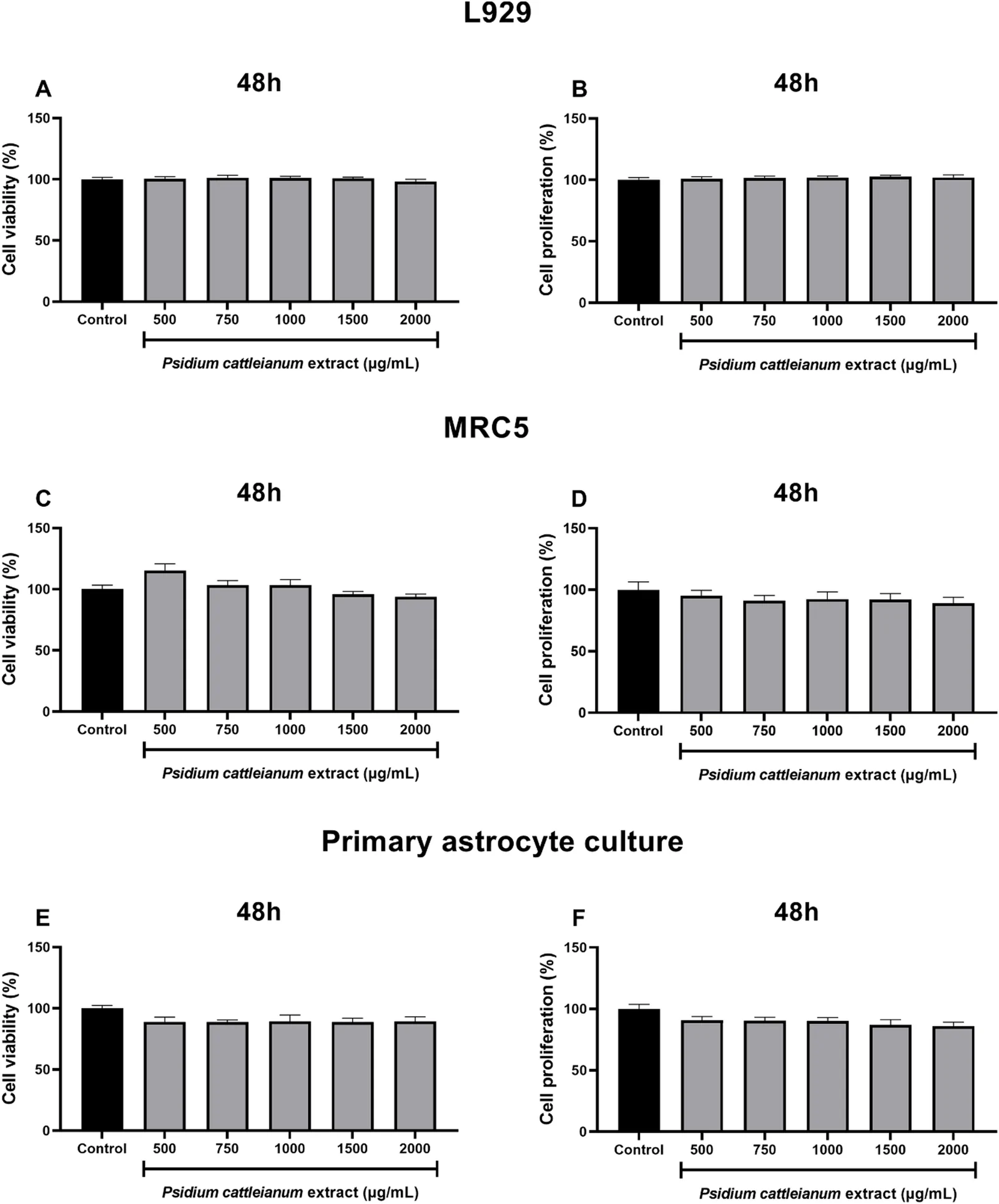
Effect of hydroalcoholic extract from yellow *Psidium cattleianum* fruits on cell viability **A**, **C**, and **E** and cell proliferation **B**, **D**, and **F** in fibroblast cell lines L929 **A**, **B**, MRC5 **C**,** D**, and primary astrocytes **E**, **F** after 48 h. Data are expressed as mean ± standard error and analyzed by one-way ANOVA with Tukey’s *post-hoc* test. Three independent experiments were performed in triplicate

The effect of YPC extract was also investigated in healthy cell lines and in a primary astrocyte culture to evaluate its potential toxicity. After the intermediate time of 48 h, no cytotoxic effect was observed in fibroblast cell lines (MRC5 and L929) or in the primary astrocyte culture (Fig. 3). This time point was selected for the evaluation of non-tumor cells because it represented an intermediate treatment window that yielded consistent and comparable dose–response patterns with those observed at 72 h in the MTT and SRB assays.

### Effect of Yellow *Psidium cattleianum* Extract on Colony Formation

**Fig. 4:**
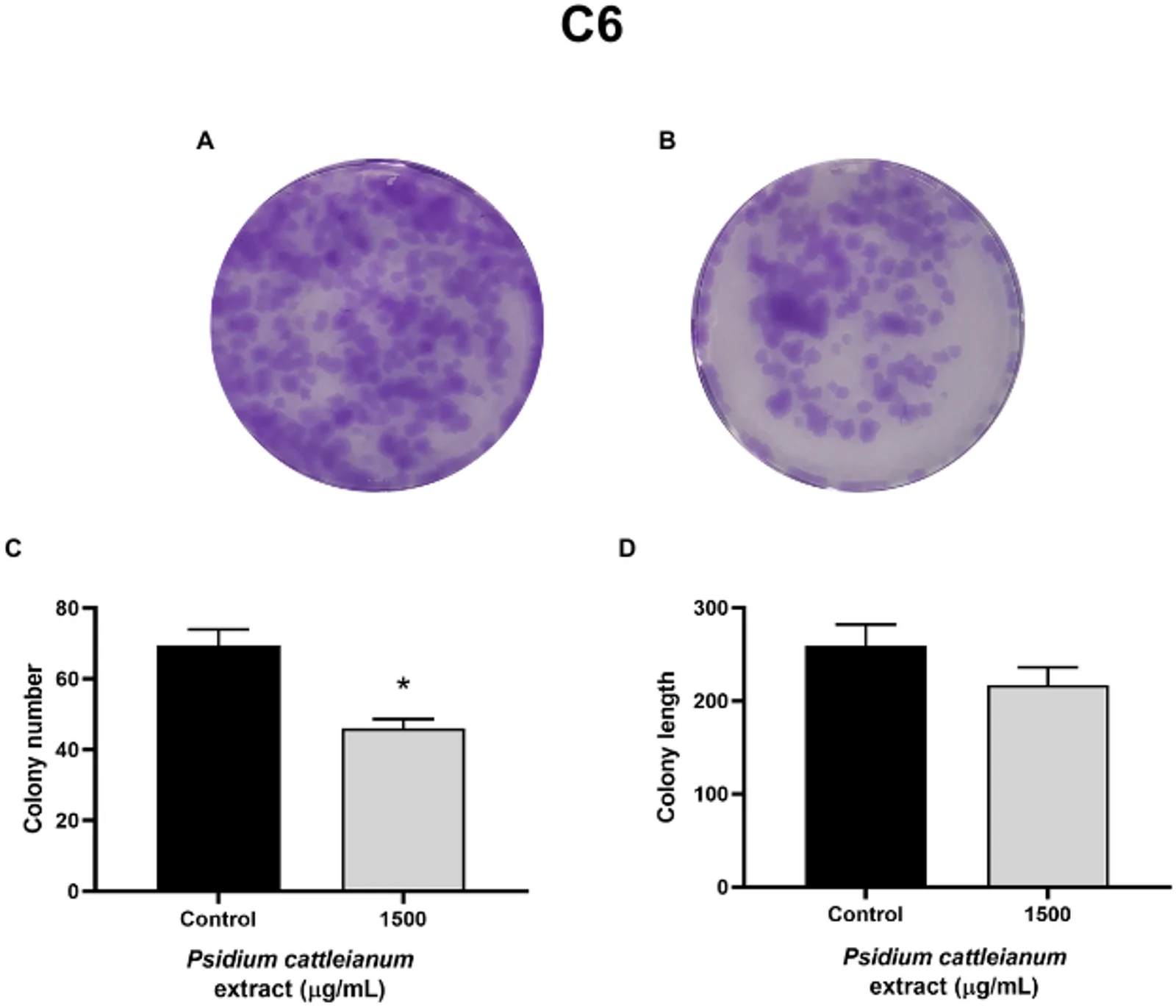
Clonogenic assay of C6 rat glioma cells: untreated **A** and treated with hydroalcoholic extract from yellow *Psidium cattleianum* fruits (1500 µg/mL) for 48 h **B**. Graphic representation of colony number **C** and colony size **D**. Data are expressed as mean ± standard error and analyzed by Student’s t-test. *p < 0.05 significantly different from the control. Three independent experiments were performed in triplicate

**Fig. 5:**
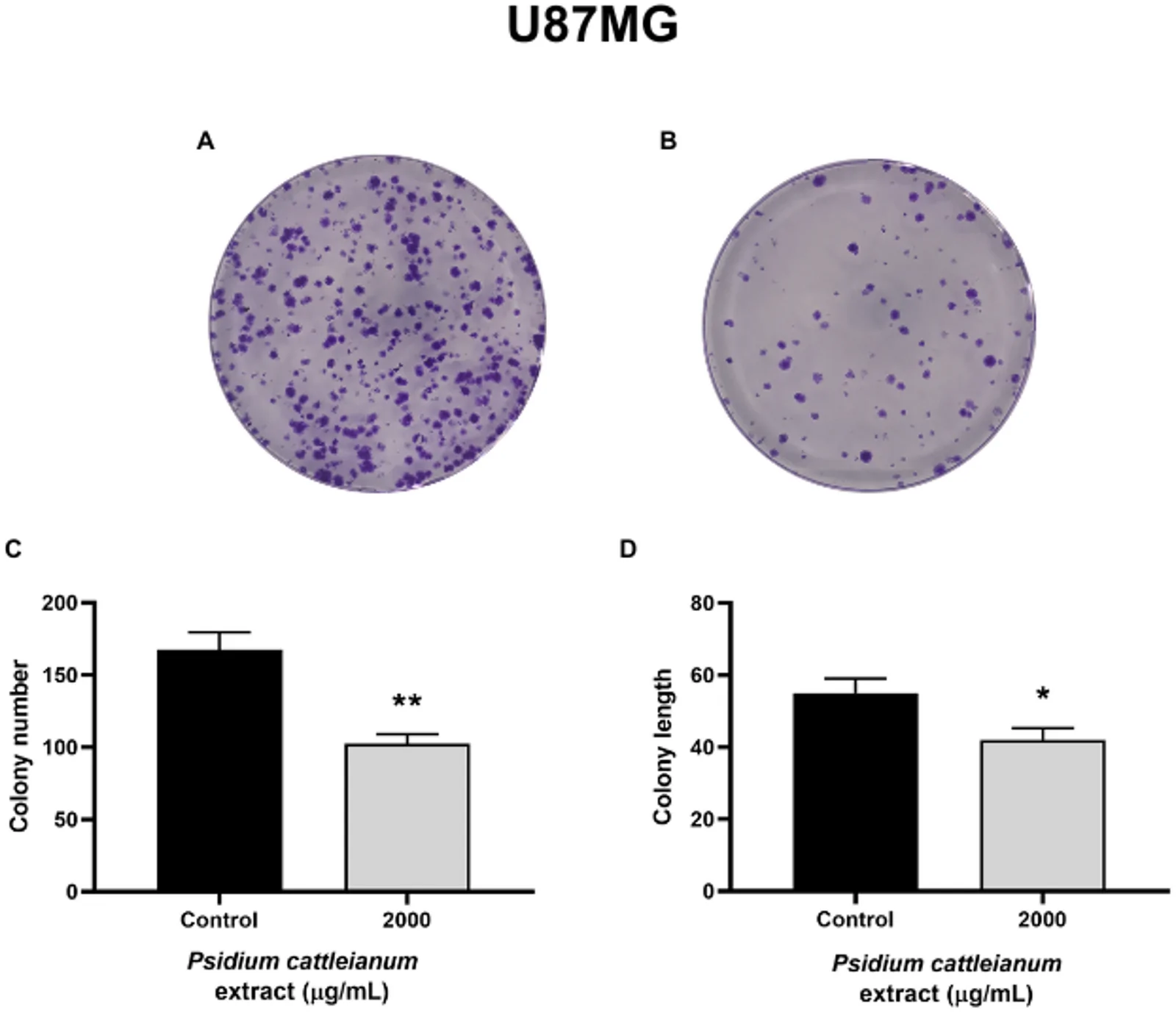
Clonogenic assay of U87MG human glioma cells: untreated **A** and treated with hydroalcoholic extract from yellow *Psidium cattleianum* fruits (2000 µg/mL) for 48 h **B**. Graphic representation of colony number **C** and colony size **D**. Data are expressed as mean ± standard error and analyzed by Student’s t-test. *p < 0.05, **p < 0.01 significantly different from the control. Three independent experiments were performed in triplicate

Treatment with YPC at a concentration of 1500 µg/mL for 48 h did not significantly reduce colony length in C6 cells (Fig. 4D). However, it reduced the number of colonies by 33.6% compared with the control (Fig. 4C). Furthermore, treatment of U87MG cells at 2000 µg/mL for 48 h reduced the number of colonies by 39% and colony length by 23.6% compared with the control (Fig. 5).

### Inhibitory Effect of Yellow *Psidium cattleianum* Extract on Cell Migration

**Fig. 6:**
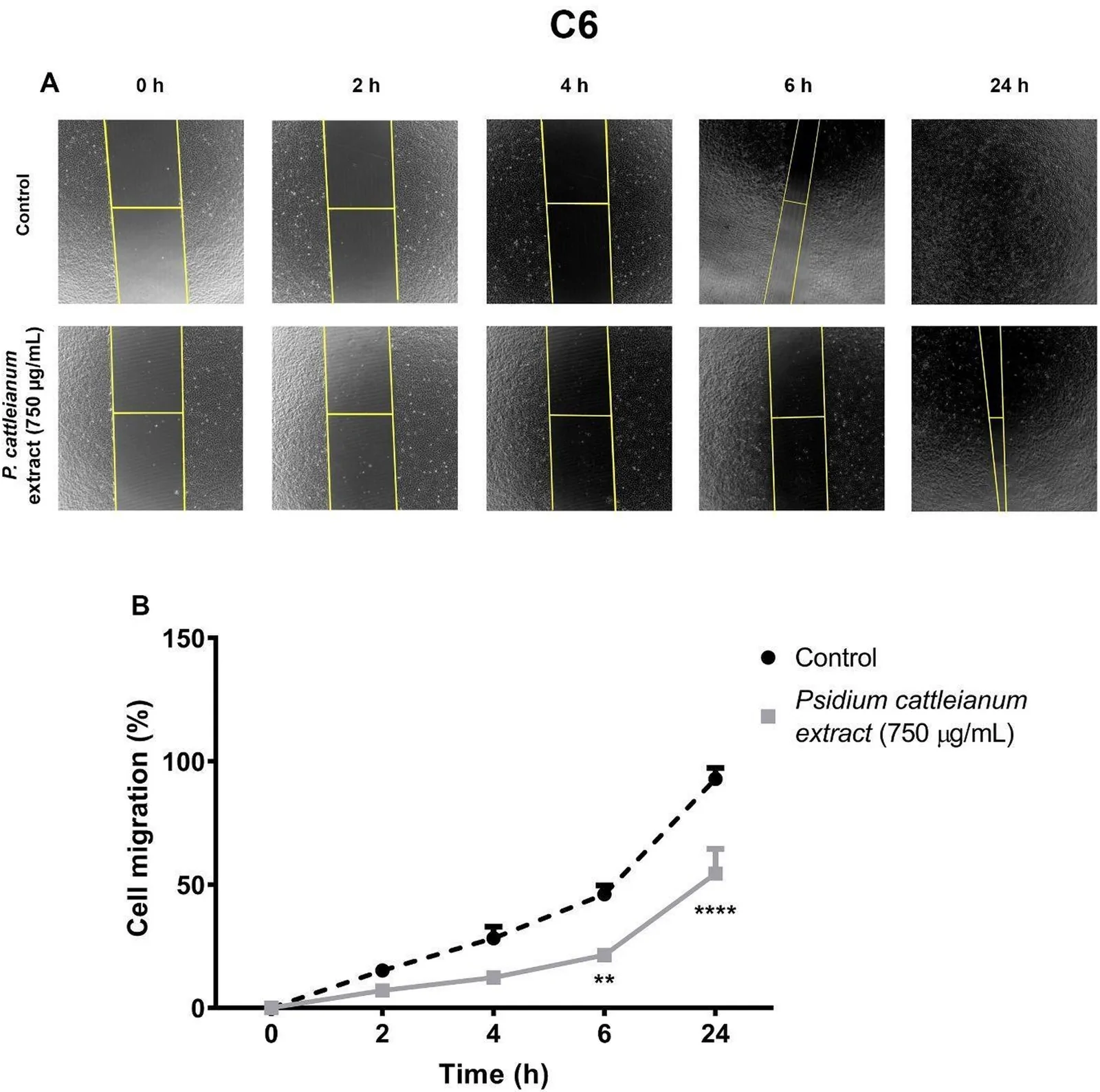
Effect of hydroalcoholic extract from yellow *Psidium cattleianum* fruits (750 µg/mL) on the migration of C6 rat glioma cells at 0, 2, 4, 6, and 24 h **A** and **B**. Data are expressed as mean ± standard error and analyzed by two-way ANOVA with Bonferroni’s *post-hoc* test. **p ˂ 0.01, ****p < 0.0001 significantly different from the control. Three independent experiments were performed in triplicate

The wound healing assay was performed to evaluate the effect of YPC on the migration of C6 rat glioma cells. Images were captured at 0, 2, 4, 6, and 24 h post-scratch (Fig. 6). YPC extract significantly inhibited cell migration, with inhibition rates of 24.8% at 6 h and 38.4% at 24 h at 750 µg/mL (Fig. 6B). The 750 µg/mL concentration was chosen as it did not show cytotoxicity on cell viability or proliferation after 24 h. This ensures that the observed reduction in wound closure reflects a genuine inhibition of cell motility and migration rather than a consequence of cell death.

### Effect of Yellow *Psidium cattleianum* Extract on Cell Death

**Fig. 7:**
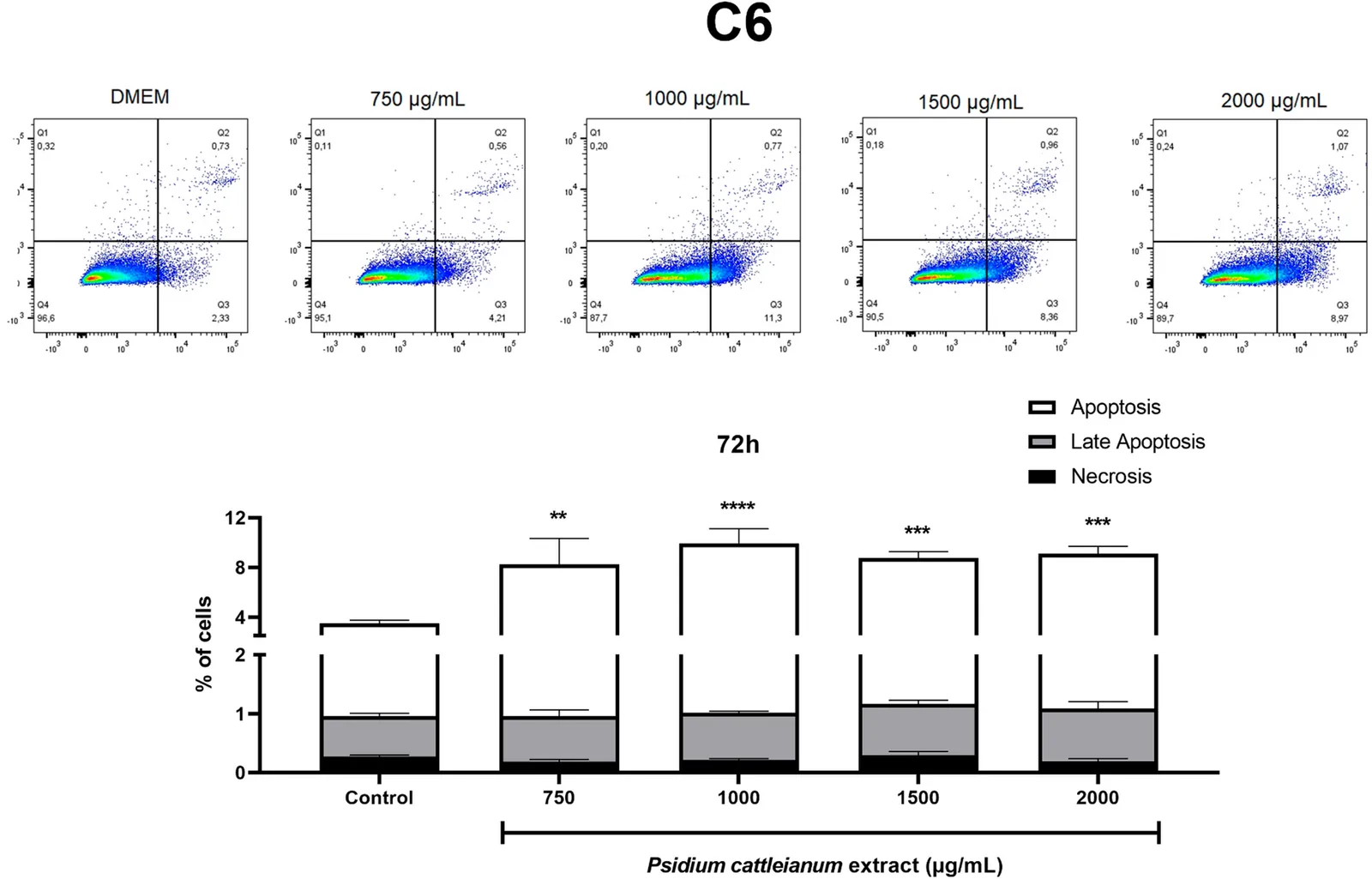
Effect of hydroalcoholic extract from yellow *Psidium cattleianum* fruits (750–2000 µg/mL) on cell death in C6 rat glioma cell line at 72 h. Data are expressed as mean ± standard error and analyzed by two-way ANOVA with Tukey’s *post-hoc* test. **p ˂ 0.01, ***p ˂ 0.001, ****p < 0.0001 significantly different from the control. Three independent experiments were performed in triplicate

To investigate a possible mechanism by which YPC extract exerted its cytotoxic effect, an Annexin V/PI assay was conducted in C6 rat glioma cells. After 72 h of treatment, an increase in apoptosis of 4.8%, 6.4%, 5.1%, and 5.5% was observed at 750, 1000, 1500, and 2000 µg/mL, respectively, compared with the control (Fig. 7).

### Yellow *Psidium cattleianum* Extract Attenuates ROS Levels in C6 and U87MG Cell Lines

**Fig. 8:**
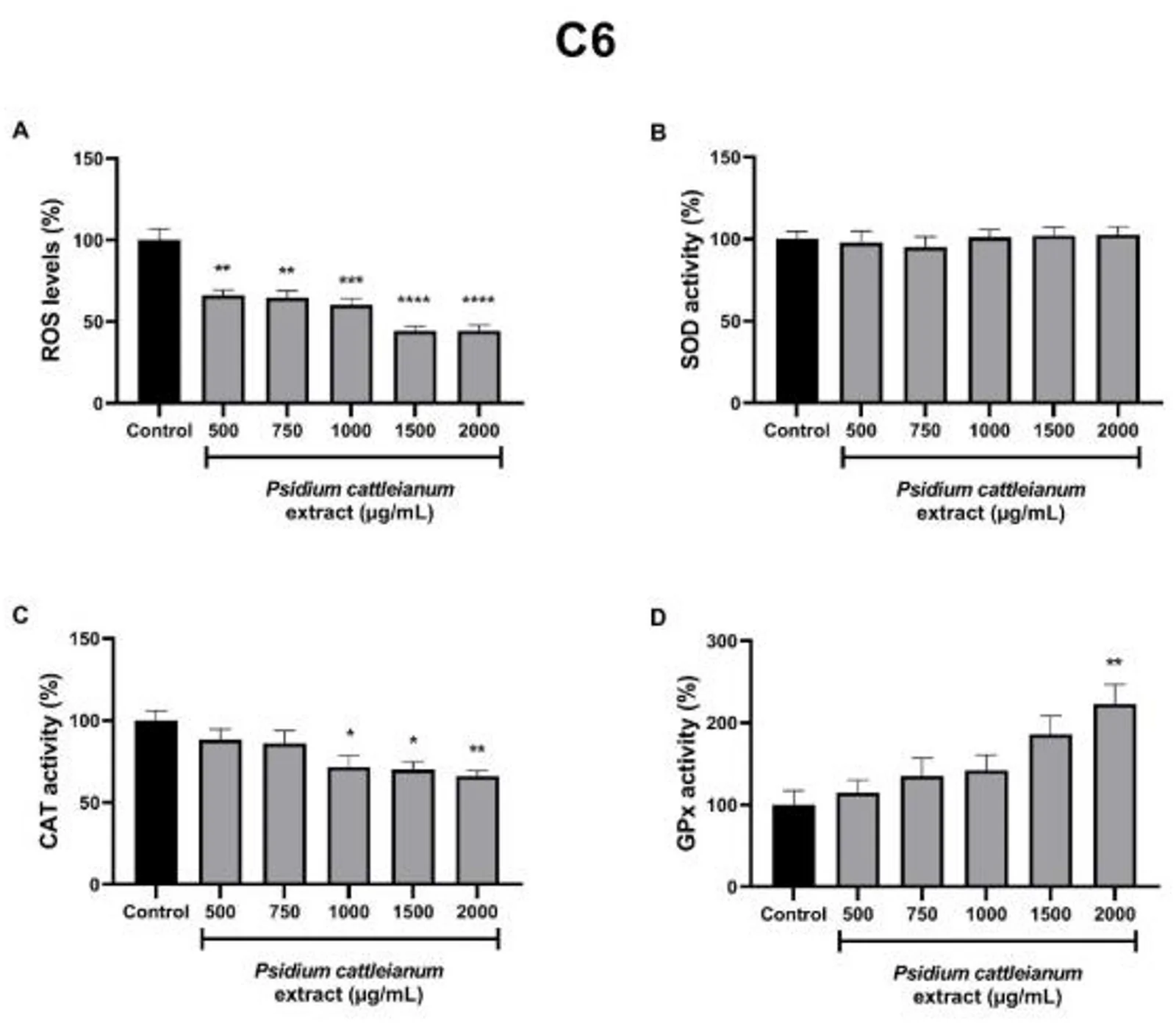
Effect of hydroalcoholic extract from yellow *Psidium cattleianum* fruits (500–2000 µg/mL) on redox equilibrium parameters in C6 rat glioma cell line after 48 h. **A** Reactive oxygen species levels (ROS); **B** superoxide dismutase activity (SOD); **C** catalase activity (CAT); **D** glutathione peroxidase (GPx). Data are expressed as mean ± standard error and analyzed by one-way ANOVA with Tukey’s *post-hoc* test. *p < 0.05, **p < 0.01, ***p < 0.001, ****p < 0.0001 significantly different from the control. Three independent experiments were performed in triplicate

**Fig. 9:**
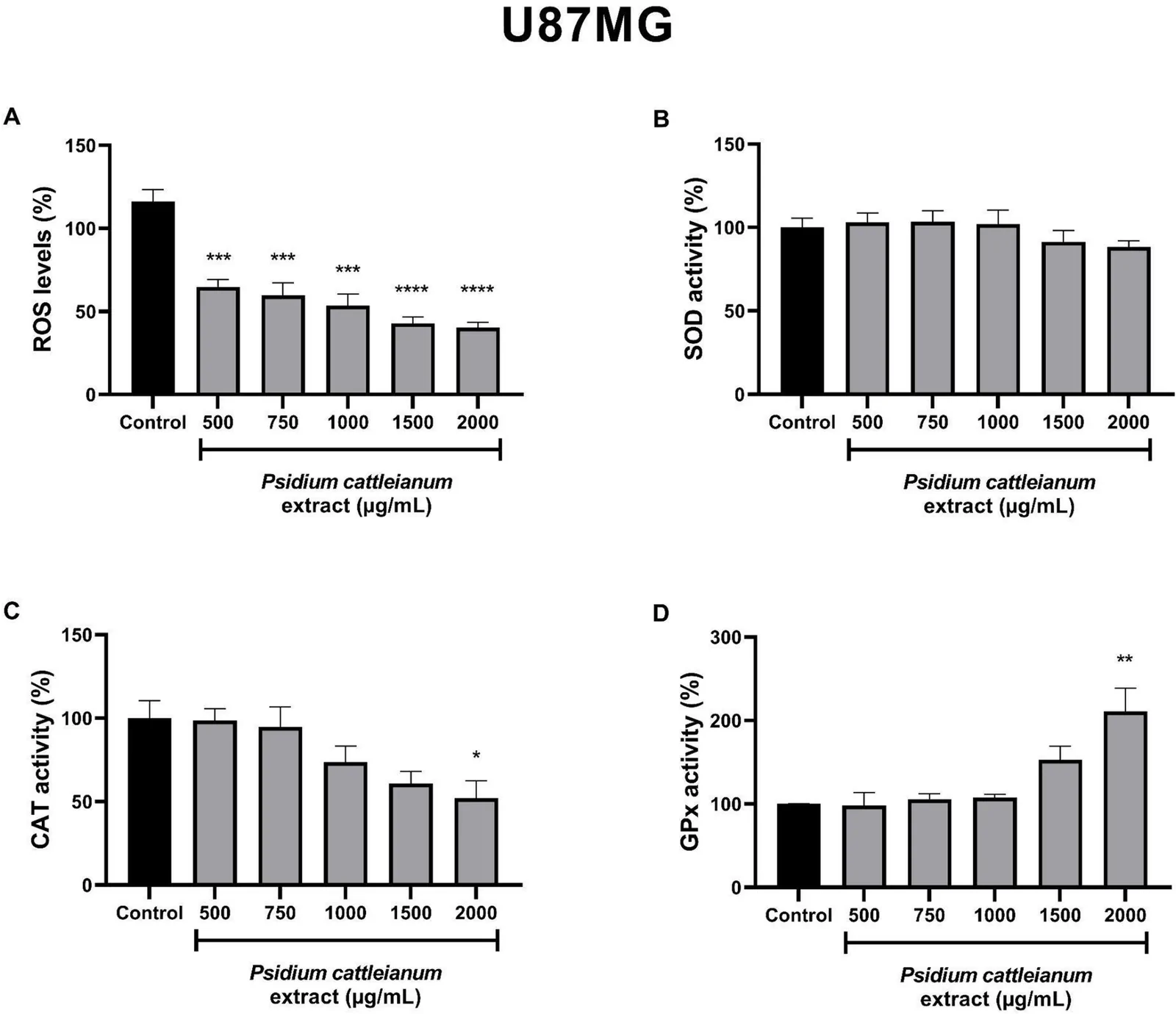
Effect of hydroalcoholic extract from yellow *Psidium cattleianum* fruits (500–2000 µg/mL) on redox equilibrium parameters in U87MG human glioma cell line after 48 h. **A** Reactive oxygen species levels (ROS); **B** superoxide dismutase activity (SOD); **C** catalase activity (CAT); **D** glutathione peroxidase (GPx). Data are expressed as mean ± standard error and analyzed by one-way ANOVA with Tukey’s *post-hoc* test. *p < 0.05, **p < 0.01, ***p < 0.001, ****p < 0.0001 significantly different from the control. Three independent experiments were performed in triplicate

To evaluate the effect of the YPC extract on redox balance parameters, the highest concentrations of the extract (500–2000 µg/mL) were used. After 48 h of treatment, a significant reduction in ROS levels was observed in the C6 cell line at all tested concentrations, by 33.8%, 35.2%, 39.8%, 55.7%, and 55.5% (Fig. 8). Similarly, ROS levels were reduced in the U87MG cell line by 51.5%, 56.5%, 62.7%, 73.5%, and 76.0% (Fig. 9). Regarding antioxidant enzyme activities, no changes were observed in SOD activity in either cell line. However, a reduction in CAT activity (Figs. 8 and 9) and an increase in GPx activity at the highest tested concentration (2000 µg/mL) (Figs. 8 and 9) were detected.

## Discussion

Despite current interventions, GBM remains an incurable tumor with poor prognosis and high recurrence [3, 4]. Thus, the search for therapies with improved efficacy and reduced toxicity against GBM remains critical. We characterized the phenolic composition of a hydroalcoholic extract from YPC fruits and evaluated its antitumor effects in C6 and U87MG glioma cells.

Previous studies have evaluated the phenolic content of YPC, both directly in the fruits and in extracts obtained with aqueous and acetonic solvents [14, 30–32]. However, to the best of our knowledge, this is the first study to identify the major phenolic compounds specifically in a hydroalcoholic extract. The phenolic composition of plant matrices is influenced by multiple factors, including genetic background, environmental conditions, cultivation practices, ripening stage, storage, and the solvent used in extraction [33]. Accordingly, phytochemical characterization of the YPC hydroalcoholic extract is relevant. The use of hydroalcoholic solutions is supported by evidence indicating that solvent mixtures promote more efficient phenolic extraction compared to single solvents, owing to differences in polarity [34], with 50–70% ethanol solutions typically optimizing flavonoid and tannin yields in plant matrixes [35].

In the present study, the main quantified phenolics in the dry YPC hydroalcoholic extract were ellagic acid (13.34 ± 0.39 µg g⁻¹), catechin (6.42 ± 0.06 µg g⁻¹), and gallic acid (4.47 ± 0.10 µg g⁻¹). Placing these quantitative results into a broader phytochemical context reveals substantial numerical variation across the literature, which is largely driven by differences in extraction procedures, matrix processing (e.g., dry extract vs. fresh fruit pulp), and the use of chemical hydrolysis. For instance, Betta et al. [31] evaluated free phenolics in unhydrolyzed yellow strawberry guava dry matter, reporting catechin as the predominant compound (480.3 to 1077.5 µg g⁻¹) alongside lower levels of gallic acid (6.2 to 7.1 µg g⁻¹ dry matter). Upon acidic hydrolysis of yellow strawberry guava dry tissue, Schulz et al. [32] reported liberated catechin levels ranging from 108.8 to 127.9 µg g⁻¹ dry matter and gallic acid from 5.3 to 15.4 µg g⁻¹ dry matter. In fresh fruit pulp extracts of yellow and red accessions, Medina et al. [14] quantified (-)-epicatechin up to 2659.5 µg g⁻¹ fresh pulp and gallic acid up to 801.0 µg g⁻¹ fresh pulp, expressing values relative to fresh biomass rather than concentrated dry extract mass. Furthermore, when analyzing concentrated dry ethanolic extracts of red *Psidium cattleianum*, Ribeiro et al. [36] observed markedly higher concentrations of ellagic acid 2213 to 3818 µg g⁻¹ extract) and gallic acid (464 to 1510 µg g⁻¹ extract).

Characterizing the phenolic composition of natural extracts enhances understanding of their mechanisms and potential therapeutic applications [37]. Phenolic compounds have demonstrated activity against highly resistant tumors such as GBM, representing a promising avenue for cancer therapy [38]. Their bioactivities, including reports of synergistic effects with TMZ [39], provide a strong rationale for investigating natural extracts as sources of multiple phenolics, whose combined effects may complement conventional therapies and help overcome challenges posed by this aggressive brain tumor. Accordingly, this study assessed the antiglioma potential of a hydroalcoholic YPC fruit extract in C6 (rat) and U87MG (human) cells. These cell lines are widely used in in vitro studies and serve as established models for investigating tumor processes, assessing cytotoxicity, and screening compounds with potential antineoplastic activity against GBM [40].

Our results showed that the YPC hydroalcoholic extract inhibited cell viability and proliferation in both cell lines, an effect likely related to its phenolic composition. Gallic acid has demonstrated antitumor effects against GBM by inhibiting tumor growth in vitro and in vivo and modulating pathways involved in tumor development and proliferation [19, 41]. Catechin has also shown antiglioma activity through induction of autophagy, cell cycle arrest, and inhibition of migration and invasion [42]. Similarly, ellagic acid inhibited GBM growth both in vitro and in vivo by inducing DNA damage and S-phase arrest [43]. The observed in vivo effects suggest that these compounds may cross the blood-brain barrier [19, 43], which is an essential consideration for the development of therapeutic strategies targeting brain cancer.

Combinations of natural compounds, such as those present in plant extracts, may exhibit additive or synergistic effects, potentially resulting in biological responses that differ from those observed for individual compounds [44]. At the highest extract concentration tested (2000 µg/mL), the estimated levels of gallic acid, catechin, and ellagic acid were 0.0526 µM, 0.0442 µM, and 0.0883 µM, respectively. These concentrations are markedly lower than those reported to exert biological effects when the corresponding isolated compounds were evaluated individually [19, 41–43]. Thus, the observed biological activity is unlikely to be explained solely by the individual contribution of these three compounds. Rather, it may result from the combined effects of multiple constituents present in the extract, including compounds that were not quantified in the present study. Additive or synergistic interactions among these constituents may also contribute to the overall biological activity; however, because such interactions were not directly investigated, this possibility cannot be confirmed based on the present findings.

The YPC extract did not affect the viability or proliferation of human (MRC5) and mouse (L929) fibroblasts, nor of primary rat astrocytes, indicating selective cytotoxicity toward glioma cells. Similarly, Medina et al. [14] reported selective cytotoxic effects of acetone and aqueous YPC extracts against MCF-7 and Caco-2 cells. More recently, Moraes et al. [16] showed that ethanolic YPC extract was non-toxic in *Caenorhabditis elegans* larvae, while extending lifespan and attenuating oxidative stress. Such findings are relevant given that conventional GBM treatment, particularly with TMZ, is associated with severe adverse effects, including myelosuppression and opportunistic infections [45]. While these results provide valuable proof-of-concept for the extract’s bioactivity, the maximal biological effects in our in vitro models were observed at the highest tested concentration (2000 µg/mL). Such high concentrations may not directly translate to realistic dietary consumption of the fruit, given the challenges of bioavailability, gastrointestinal metabolism, and systemic absorption. Furthermore, although non-toxic in our preliminary assays, validating the safety profile of such high concentrations will require comprehensive toxicity and pharmacokinetic studies in animal models before considering therapeutic applications.

The invasive growth pattern of GBM in surrounding brain tissues significantly contributes to treatment resistance and low patient survival [6]. Identifying compounds that reduce tumor cell growth and motility is therefore highly relevant. In this study, YPC extract attenuated growth and colony formation in C6 and U87MG cells and inhibited migration in C6 cells. These effects align with previous reports in which gallic acid and catechin reduced colony formation and migratory capacity in glioma cells [41, 42]. Tumor invasion and migration in GBM involve degradation of the extracellular matrix, primarily mediated by matrix metalloproteinases (MMPs), which facilitate infiltration and tumor progression [46]. Ellagic acid suppressed MMP-2 and MMP-9 expression in glioma xenografts [43], and an extract of *Butia odorata*, another yellow fruit, reduced MMP-2 levels in C6 cells [20]. Thus, these findings suggest that YPC extract may inhibit GBM migration, potentially through regulation of MMP expression.

The YPC extract may slightly promote apoptotic features in C6 glioma cells, as indicated by the modest increase (5–6%) in apoptotic rates observed in our assays. Apoptosis is characterized by a series of biochemical and morphological events that lead to programmed cell death without releasing intracellular contents into the extracellular environment [47]. It is a critical physiological process where activation in response to DNA damage eliminates potentially harmful precancerous cells and prevents tumor growth [48]. Unlike necrosis, which is uncontrolled and triggers inflammation, apoptosis is a regulated process [47]. Although these preliminary findings suggest a potential pro-apoptotic effect, further studies, such as evaluating caspase activation (e.g., caspase-3, -8, and − 9) and DNA fragmentation, are required to definitively confirm the induction of apoptosis. In addition, YPC extract might influence apoptosis-related pathways, possibly modulating the expression of antiapoptotic proteins like Bcl-2, as ellagic acid has been shown to reduce Bcl-2 levels in a glioma xenograft model [43].

Finally, our results suggest that the YPC extract reduced the ROS levels in both U87MG and C6 cell lines. ROS is a broad concept, encompassing oxygen (O₂) free radicals and certain non-radical derivatives, including hydrogen peroxide (H₂O₂) [8]. In the present study, ROS levels were assessed using the DCF assay, which detects oxidants in both the mitochondria and the cytosol of cells [49]. Although it does not differentiate between specific ROS and is more sensitive to H₂O₂ than to free radicals, it remains a widely employed method, as H₂O₂ participates in the Fenton reaction to generate hydroxyl radical (•OH), which can oxidize proteins, lipids, and DNA [8, 49].

In view of this, the observed reduction in ROS levels likely reflects lower intracellular H₂O₂. Since SOD levels remained unaltered, this may suggest that the conversion of O₂^•⁻^ to H₂O₂ was unaffected. However, it is important to consider that the method used for SOD detection is non-specific and, due to sample preparation, likely reflected predominantly the activity of the cytosolic isoform, SOD1 [28]. Therefore, an alternative hypothesis is that the activity of the mitochondrial isoform, SOD2, may have increased without being detected, thereby contributing to O₂^•⁻^ dismutation. This interpretation is consistent with studies reporting increased SOD2 activity or expression without corresponding changes in SOD1 [50, 51]. Regarding CAT, the reduction observed in this study suggests that other antioxidant systems may be compensating for H₂O₂ degradation, such as the GPx enzyme, for which an increase was observed.

While antioxidant capacity is well-established in chemoprevention, the functional impact of ROS attenuation in established cancer cells is more nuanced. Cancer cells often operate under elevated baseline oxidative stress to drive pro-survival, migratory, and proliferative signaling cascades [7, 8]. In this context, the significant reduction of intracellular ROS levels and increase in GPx activity observed following YPC extract treatment may suppress these redox-dependent pro-tumoral pathways. However, since excessive ROS accumulation can also trigger oxidative cell death, the reduction in ROS observed in the present study should be interpreted primarily as an indicator of altered redox homeostasis rather than as evidence of a direct causal role in the cytotoxic effects of the YPC extract. Further mechanistic studies investigating specific redox-sensitive signaling pathways, such as Nrf2, MAPK/ERK, and Akt, will be necessary to determine whether ROS modulation contributes directly to the antiglioma effects of the YPC extract or represents a secondary consequence of its biological activity.

Here, we demonstrate the potential anti-glioma effect of YPC; however, future studies are needed to further elucidate the extract’s effects, given that the present study, despite its promising results, has certain limitations that should be addressed in future work. First, cell migration and death pathways were evaluated primarily in C6 cells; future studies should expand these assays to human GBM lines (e.g., U87MG and primary patient-derived cells) to confirm cross-species translation. Second, while enzymatic activities were established here, subsequent investigations should evaluate the gene and protein expression of specific isoforms (e.g., SOD1, SOD2, and CAT) alongside the Nrf2/HO-1 pathway, quantify oxidative damage markers (such as malondialdehyde and protein carbonyls), explore potential synergistic interactions with TMZ, and characterize alternative cell death modalities (e.g., caspase activity and necroptosis). Finally, as this study was conducted entirely in vitro, it lacks the physiological complexity of tumor-microenvironment interactions; thus, evaluating YPC extract efficacy and bioaccessibility in orthotopic GBM animal models will be a critical next step before clinical consideration. Nevertheless, these in vitro findings provide a necessary baseline to guide these future translational studies.

**Table 1:** Phenolic content (µg g^− 1^) of hydroalcoholic extract of yellow *Psidium cattleianum* fruits

Compounds	m/z ([M-H]^-^)	Rt (min)	Content (µg g^-1^)
*Phenolic acids*			
Caffeic acid	179.0350	8.47	0.45±0.01
Chlorogenic acid	353.0867	13.63	1.27±0.03
Gallic acid	169.0132	2.27	4.47±0.10
*p*-Coumaric acid	163.0401	9.32	0.39±0.05
*Hydrolysable tannin derivatives*			
Ellagic acid	300.9978	15.61	13.34±0.39
*Flavonoids*			
(+)-Catechin	289.0718	7.79	6.42±0.06
Kaempferol	285.0393	17.68	0.08±0.00
Quercetin	301.0354	16.85	2.22±0.03

*m/z* ([M-H]^-^): mass-to-charge ratios; Rt: retention times. Results are expressed in µg g^-1^ of dry weight, as mean ± standard deviation of four replicates

## Conclusions

The hydroalcoholic extract from YPC fruits was found to contain ellagic acid, catechin, and gallic acid as major constituents. The extract selectively inhibited proliferation and viability in C6 and U87MG glioma cells without affecting healthy cells, demonstrating a targeted cytotoxic effect. It also impaired colony formation and reduced ROS levels in both cell lines, and reduced migration in C6 cells. Collectively, these results position the hydroalcoholic YPC fruit extract as a promising candidate for further investigation as a complementary strategy in GBM therapy.

## Data Availability

The data that support the findings of this study are available from the corresponding author upon reasonable request.
